# Training for Improvement of communication between patient psychiatrists and families

**DOI:** 10.1192/j.eurpsy.2025.2383

**Published:** 2025-08-26

**Authors:** S. Strkalj Ivezic, D. Bošnjak Kuharić, M. Batković, A. Papić

**Affiliations:** 1 School of Medicine, University Psychiatric Hospital Vrapče; 2 University Psychiatric Hospital Vrapce, Zagreb; 3 Dubrovnik County Hospital, Dubrovnik; 4 Department for psychotic disorders- male, University Psychiatric Hospital Vrapce, Zagreb, Croatia

## Abstract

**Introduction:**

Although the collaborative partnership of the psychiatrist, patient and family is vital to recovery from a mental disorder, many patients and their family members are not satisfied with the communication with the psychiatrist. They believe that they are not actively involved in creating a treatment plan and that they do not have enough information about the diagnosis and treatment.

**Objectives:**

Objective of the study is to improve the communication skills of young psychiatrists for collaboration with patients and their families.

**Methods:**

Recognising the pressing need to address dissatisfaction with communication, we implemented the EUFAMI Prospect Common Ground three-way communication program, which we adapted for our study. We measured the increase in communication skills using a self-assessment questionnaire.

Participants: three groups of patients diagnosed with severe mental illness, family members, and young psychiatrists have been working in small groups separately and together in large groups.

**Results:**

Our study’s results are promising. The majority of participants expressed satisfaction with the training method. Their feedback highlighted acquiring new communication skills, which they believe will strengthen the collaborative relationship between patients, family members, and psychiatrists.

**Conclusions:**

A training program for improving communication skills for young psychiatrists proves to be useful for improving the creation of a therapeutic relationship of cooperation with patients and their family members.

**Disclosure of Interest:**

None Declared

